# Single-bundle ACL reconstruction with and without extra-articular reconstruction: evaluation with robotic lower leg rotation testing and patient satisfaction scores

**DOI:** 10.1007/s00167-015-3772-8

**Published:** 2015-09-04

**Authors:** Thomas Branch, Frédéric Lavoie, Christian Guier, Eric Branch, Timothy Lording, Shaun Stinton, Philippe Neyret

**Affiliations:** University Orthopedics, Decatur, GA USA; Department of Orthopedic Surgery, Hôpital Notre-Dame, CHUM, Montréal, QC Canada; Private Practice, Jacksonhole, WY USA; Miami, FL USA; Frankston Hospital, Frankston, VIC Australia; ArthroMetrix LLC, 441 Armour Place NE, Atlanta, GA 30324 USA; Department of Orthopaedic Surgery, Centre Albert-Trillat, Hôpital de la Croix-Rousse, Lyon, France

**Keywords:** Rotational laxity, Robotic knee testing, Extra-articular augmentation, ACL reconstruction, Knee laxity

## Abstract

**Purpose:**

The purpose of this study was to compare the biomechanical characteristics and patient outcomes after either isolated intraarticular ACL reconstruction or intraarticular reconstruction with lateral extra-articular tenodesis. In addition, we aimed to evaluate biomechanical parameters of the entire uninjured, contralateral knee as a baseline during the analysis.

**Methods:**

Eighteen patients were evaluated at an average of 9.3 years after ACL reconstruction. Twelve patients had an intraarticular reconstruction (BTB), and six had an additional lateral extraarticular procedure (BTB/EAR). Patients were selected for the additional procedure by the operating surgeon based on clinical and radiological criteria. At the time of review, each patient was assessed using subjective patient questionnaires, manual laxity testing, and instrumented laxity testing. Each knee was also evaluated using a robotic lower leg axial rotation testing system. This system measured maximum internal and external rotations at 5.65 Nm of applied torque and generated load deformation curves and compliance data. Pointwise statistical comparisons within each group and between groups were performed using the appropriate paired or unpaired *t* test. Features were extracted from each load deformation curve for comparative analysis.

**Results:**

There were no significant differences between the two groups with respect to the patient satisfaction scores or to laxity testing (manual or instrumented). Robotic testing results for within-group comparisons demonstrated a significant reduction in maximum external rotation (8.77°) in the reconstructed leg when compared to the healthy leg (*p* < 0.05) in the BTB/EAR group, with a non-significant change in internal rotation. The slope of the curve at maximum internal rotation was also significantly greater in the reconstructed legs for the BTB/EAR group (*p* < 0.05), indicating reduced endpoint compliance or a harder endpoint. Finally, the leg that received the extra-articular tenodesis had a trend towards a reduced total leg axial rotation. Conversely, patients in the BTB group demonstrated no significant differences between their legs. For between-group comparisons, there was a significant increase in maximum internal rotation in the healthy legs in the BTB/EAR group compared with the healthy legs in the BTB group (*p* < 0.05). If the injured/reconstructed legs were compared, the significant difference at maximum internal rotation disappeared (*p* < 0.10). Similarly, the healthy legs in patients in the BTB/EAR group had a significantly more compliant or softer endpoint in internal rotation, greater maximum internal rotation, and more internal rotation at torque 0 in their healthy legs compared with the healthy legs in the BTB group (*p* < 0.05). These same differences were not noted in the reconstructed knees. The only identifiable significant difference between the injured/reconstructed legs was rotation at 0 torque (*p* < 0.05).

**Conclusions:**

In this group of patients who were at an average of 9 years from surgery, the addition of a lateral extra-articular reconstruction to a standard bone–tendon–bone intraarticular ACL reconstruction does reduces internal rotation of the tibia with respect to the femur when compared to intraarticular reconstruction alone. It appears that the selection process for inclusion into the BTB/EAR group included an increase in total axial rotation of the healthy knee during the examination along with a decrease in endpoint stiffness at maximum internal rotation.

**Level of evidence:**

II.

## Introduction

One of the first surgical techniques for managing the knee instability caused by the loss of the anterior cruciate ligament (ACL) was the lateral extra-articular reconstruction (EAR). Used alone, this technique had an unacceptable rate of long-term patient satisfaction [1, 12, 17, 19, 20]. What followed was the development of intraarticular ACL reconstruction, classically, the bone–tendon–bone (BTB) reconstruction. Unfortunately, failure rates for intraarticular ACL reconstruction have been reported to be up to 20 % or more in some studies [2, 6, 10, 11, 14, 15]. In particular, biomechanical studies have found that rotational control is not well restored after ACL reconstruction [7, 13, 19, 22]. In an attempt to surgically manage this loss of rotational control, new intraarticular reconstructions using double and even triple bundles were designed. Subsequently, there was a movement towards the use of lateral EAR in addition to intraarticular reconstruction in an attempt to better control rotation [3, 4, 8, 21]. However, early studies showed that results with the combined procedure were not improved over results from intraarticular reconstruction alone, and the technique was not widely used [10, 14, 16]. More recent studies have reported better results in terms of improved rotational stability and subjective patient scores through use of combined extra-articular and intraarticular repair with no increased rate of complications and radiologic signs of osteoarthritis or arthrofibrosis [5, 18, 19]. These studies focused on patients with severe instability, high-level athletes, or revision procedures.

ACL reconstructions are performed with the intent of impacting knee biomechanics. An uninjured knee has a normal amount of ‘joint play’, or in other words, a normal amount of small motions between tibia and the femur that are restricted by the tensioning of uninjured ligaments. Loss of the ACL results in a change in ‘joint play’ with increased anterior translation and internal rotation. In addition, the anterior translation of the lateral tibial plateau is more than in the medial tibial plateau in an isolated ACL tear. The focus of this paper was to determine whether the addition of a lateral EAR to the traditional intraarticular ACL reconstruction could better restore the three-dimensional change in ‘joint play’ caused by loss of the ACL (i.e. manage the increased internal rotation).

The purpose of this study was to compare the biomechanical characteristics and patient outcomes after either isolated intraarticular ACL reconstruction or intraarticular reconstruction with lateral extra-articular tenodesis in the knee. In addition, we aimed to evaluate biomechanical parameters of the entire uninjured, contralateral knee as a baseline during the analysis.

We hypothesized that the addition of the EAR would better restore control of internal rotation compared with the isolated intraarticular ACL reconstruction. Furthermore, we hypothesized that there would be biomechanical characteristics of the healthy leg present in a single-surgeon ACL reconstruction cohort that would correlate with the clinical decision to perform a lateral extra-articular procedure, such as markedly increased internal rotation and a much softer internal rotation endpoint compliance compared with the average healthy leg in patients who did not receive the EAR.

## Materials and methods

### Patient selection

After obtaining informed consent, 18 patients who had undergone previous intraarticular BTB ACL reconstruction were retrospectively reviewed. Ethics approval was not required at the institution at the time of the study. All surgeries were performed between January 1998 and May 1999. All patients with the exception of one underwent a unilateral ACL reconstruction. The term ‘healthy’ was chosen to describe the asymptomatic, uninjured knee. One patient had bilateral ACL procedures performed and was not included in the analysis, but is presented as an example in the discussion.

At the time of surgery, one surgeon selected patients to undergo either an intraarticular BTB ACL reconstruction (BTB group) or an intraarticular BTB ACL reconstruction with additional lateral EAR (BTB/EAR group). The selection criteria were based upon the patient history, physical examination, and radiographic findings prior to surgery. The criteria for placement of patients into the BTB/EAR group included at least one of the following: previous medial meniscectomy, a large amount of anterior tibial translation (10 mm on stress X-rays or 6 mm in comparative monopod stance), a long delay in undergoing surgery, the presence of a level 3 pivot shift, and participation in strenuous sporting activities (soccer, rugby, handball, basketball, volleyball).

### Surgical technique

**Fig. 1:**
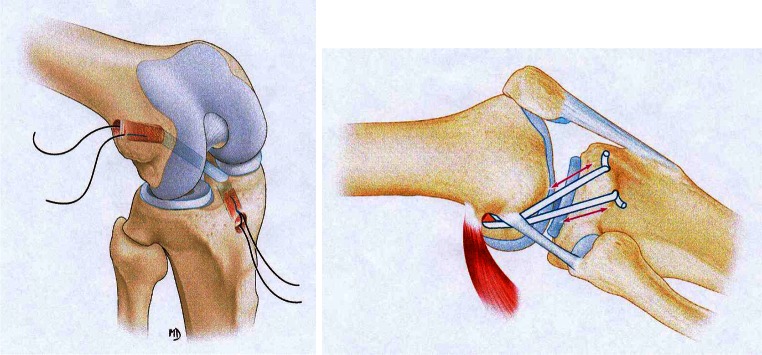
BTB reconstruction (*left*) and lateral extra-articular reconstruction (*right*)

The ACL reconstruction technique was the same for both groups and used the middle third of the patellar tendon with two bone blocks (Fig. 1). The graft was passed in an anterograde fashion, with press-fit fixation in the femur and interference screw fixation in the tibia. The extra-articular augmentation consisted of a gracilis tendon autograft, which was passed through a drill hole in the femoral bone block prior to impaction. The two free limbs were then passed under the lateral collateral ligament and attached in bone tunnels on either side of Gerdy’s tubercle, with the knee in neutral rotation and flexed to 30° (Fig. 1).

### Subjective outcome scores and manual physical examination

At review, each patient completed three questionnaires: the Knee Injury and Osteoarthritis Outcome Score (KOOS), the International Knee Documentation Committee subjective score (IKDC), and a modified visual analogue scale (VAS). Each patient was examined by two separate orthopaedists who performed manual laxity tests (Lachman–Trillat and pivot shift) and instrumented laxity tests (KT-1000 performed at 67, 89, 133 N, and manual maximum force). All subjective manual tests, including the pivot shift, Lachman, anterior drawer, range of motion, and KT-1000 tests, were performed in a randomized order by the two orthopaedic surgeons, who were blinded to the surgery as the surgical incision used in both surgical procedures was identical. Neither examiner was the surgeon of record. All procedures were performed by a single author. Finally, subjects were examined using a robotic tibial axial rotation testing system.

### Robotic testing

**Fig. 2:**
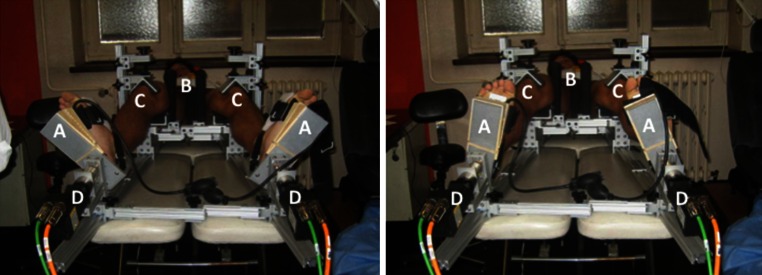
Robotic lower leg axial rotation system showing a patient whose feet are strapped into footplates (*A*), with both femurs stabilized using distal femoral posts (*B*), and both patellae locked into the trochlear grove with clamps (*C*) as torque is applied through the use of servomotors (*D*) during external rotation testing (*left*) and internal rotation testing (*right*)

The robotic knee testing system consisted of two servomotors designed to apply up to 5.65 Nm of torque about the centre of rotation of the tibia. Patients were positioned supine in the device, with both knees flexed to 30° and the second toe perpendicular to the floor (Fig. 2). The position of the toe was verified using a digital goniometer referencing the earth. The distal femur was positioned on a pad 0.1 m proximal to the joint line. One medial and one lateral post were used to rotationally position the distal femur, such that the patella rested comfortably under a patella clamp. The two posts were then brought together to clamp the distal femur, thus limiting perturbation of the femur during internal and external rotation. The patella clamp was engaged with 178 N of force to effectively anchor the patella in the trochlea of the femur and the distal femur to the posterior pad. The tibia was abducted or adducted with respect to the femur to ensure the knee rested in a neutral varus/valgus position. The patient’s feet were attached to footplates that were mounted to the servomotor system. The heel rested in a padded V to anchor and centre the heel. The centre of rotation of the lower leg was then taken to be 2.5 cm anterior to the heel at the plantar surface of the foot. The forefoot was strapped to an L plate that extended superiorly on the medial aspect of the foot. The foot was then maximally dorsiflexed by inflating a small inflatable air bladder between the sole and the plate to 60 mm Hg to limit foot and ankle motion during rotational testing.

Both extremities were rotated at the same time into external rotation followed by rotation into internal rotation. The motors rotated each leg until a peak torque of 5.65 Nm was achieved, at which point the direction of rotation was reversed. Three pre-conditioning cycles were performed, followed by three test cycles with data recording. Rotation of the leg was measured in degrees using integrated optical encoder counts in the servomotor. Current was continuously measured and converted into Nm via an on-board computer programme.

### Data analysis

Data was accumulated by Microsoft Excel and VBA, with accuracy to 0.01° and 0.001 Nm of torque as defined by the servomotors. No filtering of any kind was performed. A hysteresis curve was constructed from the three test cycles, with torque on the *y*-axis and rotation on the *x*-axis. Using the loaded portion of the hysteresis curve for each cycle, a third-order polynomial fit of the data was used for analysis. Once fitted, the curve was interpolated for a standard set of 500 torque points between −5.65 Nm (External Rotation) and +5.65 Nm (Internal Rotation). No averaging or registration was applied to the data.

Mean load deformation curves were constructed using the pointwise mean (i.e. the mean for each of the 500 torque points) of each group along with the pointwise standard error of the mean (SEM). The third full load deformation curve was considered representative of each patient. Pointwise statistical comparison was performed using the appropriate paired or unpaired *t* test.

Features were identified for each load deformation curve and were extracted for comparative analysis. The slope of the load deformation curves was reported as a percentage, with a 45° slope reported as 100 %. A higher percentage change or steeper slope represents a less compliant leg (stiffer), whereas a lower percentage change or less steep slope would represent a more compliant leg (looser) in response to rotational torque.

Other extracted features included maximum external rotation, maximum internal rotation, position of rotation at a system torque of 0 Nm (or torque zero), and the amount of play at torque zero. The amount of play at torque zero was determined by the width of the hysteresis curve, i.e. the rotation at torque zero as the leg was externally rotated minus the rotation at torque zero as the leg was internally rotated.

This paper attempts to gain knowledge of the rotational characteristics of the knee by studying the rotational characteristics of the entire lower leg. The foot–ankle connection has more rotation than the tibia–femur connection such that when rotating the lower leg from the foot, the foot moves until the ankle is locked and then the tibia moves until it reaches the torque threshold of its connection with the femur. When rotated in the opposite direction, the foot must move through a significant amount of rotation before locking the ankle again and moving the tibia.

When the whole leg is studied, special considerations must be made. The tibia is an intercalary bone which sits between the talus and the femur. The position of this bone is dependent upon the two bones on either side of it and the muscles/ligaments that connect the three bones together. There is no absolute or natural position of the tibia as its position depends upon the system as a whole. It is has a sense of freedom to position itself wherever its proximal and distal connections influence it. Thus, when a surgery is performed between two bones, the initial or resting relationship between the two bones is affected. The tibia and the fibula are connected to each other with ligaments throughout their length and that motion between the bones is extremely limited.

### Statistical analysis

Statistical analysis was performed using a custom R programme (R Foundation for Statistical Computing, Vienna, Austria) to utilize simple functional data analysis (FDA) with pointwise *t* testing across generated curves. Endpoint features were evaluated using standard sample-based statistical analysis. Initially, the results were categorized by group. Each group was further divided into reconstructed leg and healthy leg. Paired data comparisons were utilized when the analysis was between limbs within a group. Unpaired comparisons were applied when limbs from the BTB group were compared to limbs from BTB/EAR group.

## Results

### Demographics

There were 12 males and five females included in the analysis. The median age was 40 years (26–48 years). The median height and weight were 1.73 m (1.6–1.83) and 70.0 kg (55–95). The time from surgery was a median of 9 years (8 years 3 months–19 years 9 months). There were 12 patients in the BTB group and five in the BTB/EAR group. No statistically significant differences existed between the groups in terms of pre-operative laxity, the development of meniscal lesions, and degenerative changes during the follow-up period. There were eight medial meniscectomies, five in the BTB group and three in the BTB/EAR group. There were four lateral meniscectomies, two in the BTB group, and two in the BTB/EAR group. Three patients had OA in at least one compartment: two in the BTB group and one in the BTB/EAR group. The median knee extension was 0° (0°–3°), and median knee flexion was 150° (135°–160°).

### Subjective outcome scores and manual physical examination

**Fig. 3:**
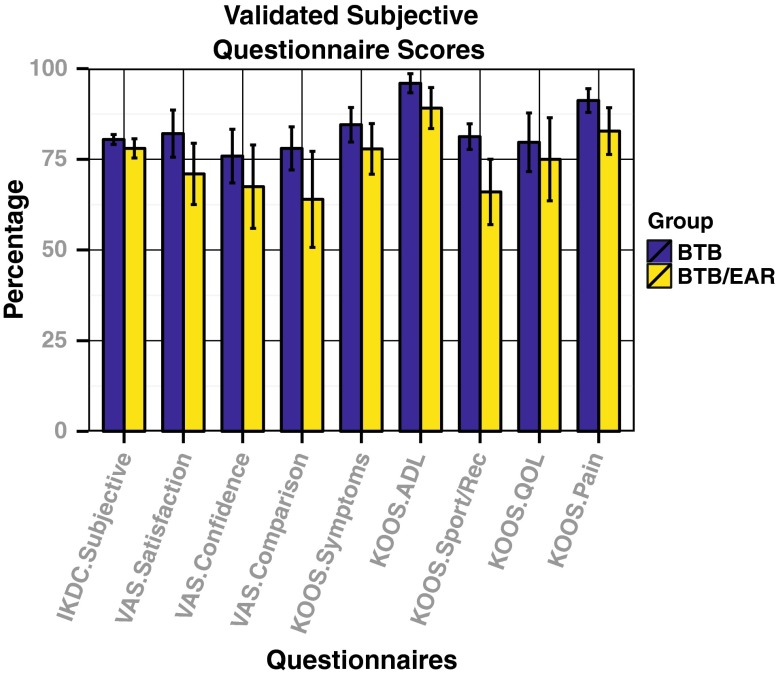
Comparison of subjective questionnaire results. No statistically significant differences were seen between the groups for any validated outcome measure

There were no significant differences between the two groups with respect to the KOOS, IKDC subjective, and VAS patient satisfaction scores (Fig. 3). VAS satisfaction scores refer to the reconstructed leg in each case. Overall patient satisfaction for both groups was high for all validated subjective outcome measures. Both groups scored >80 on the IKDC subjective score. All patients in both groups were satisfied with their healthy leg.

**Fig. 4:**
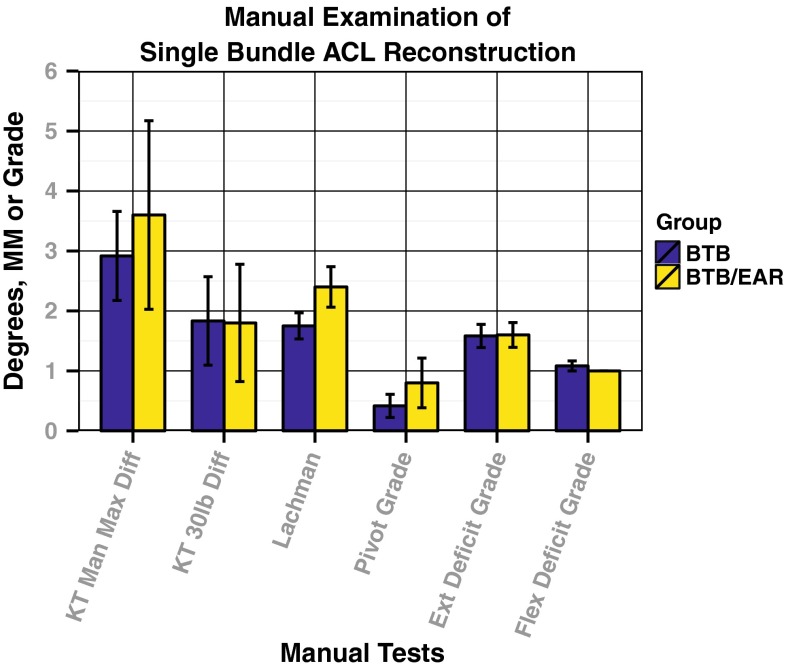
Comparison of manual physical examination results. No statistically significant differences were seen between the groups for any manual or instrumented test result

Similarly, there were no statistically significant differences between the two groups when comparing manual laxity test or instrumented laxity tests (Fig. 4). All patients had less than a 3-mm side-to-side difference at 133.5 N on the KT-1000. The mean pivot shift grade was less than grade 1. In addition, there was no significant correlation between pivot grade and any validated subjective outcome measure. There was full agreement between the two examining physicians when reporting the results of the manual examination.

### Robotic testing results

#### Within-group comparison

**Fig. 5:**
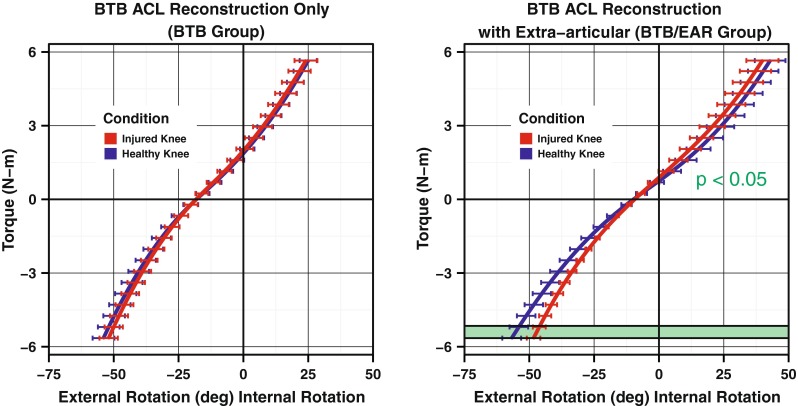
Load deformation curves for the reconstructed versus healthy leg in each group

**Fig. 6:**
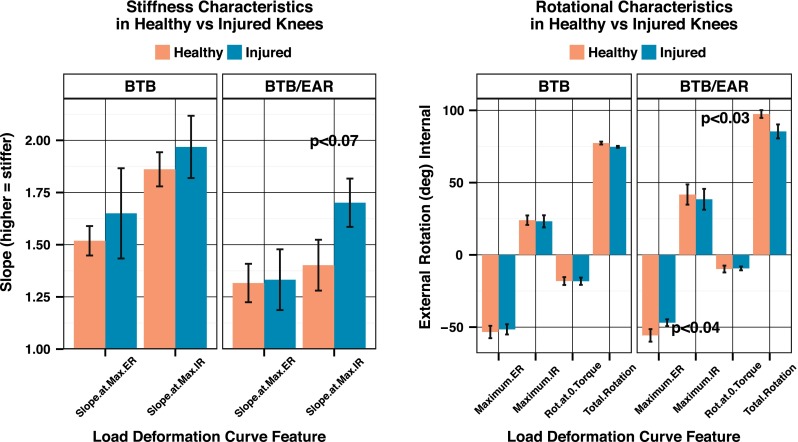
Comparison of single features of the load deformation curves was only significant in the BTB/EAR group. The BTB group showed no significant differences between the healthy and injured legs

The rotational load deformation curves generated by the robotic testing device demonstrated significant differences in rotational laxity when comparing the reconstructed leg and the healthy leg in patients in the BTB/EAR group (Fig. 5). There was a significant reduction in maximum external rotation in the reconstructed legs in the BTB/EAR group of 8.77° compared with the healthy legs (*p* < 0.04), with a non-significant change in internal rotation. The slope of the curve at maximum internal rotation showed a trend towards a stiffer endpoint in the reconstructed legs for the BTB/EAR group (*p* < 0.07) (Fig. 6). Finally, the reconstructed legs in the BTB/EAR group had reduced total axial rotation (*p* < 0.03). Conversely, patients in the BTB group demonstrated no significant differences between their healthy and reconstructed legs (Fig. 5).

**Fig. 7:**
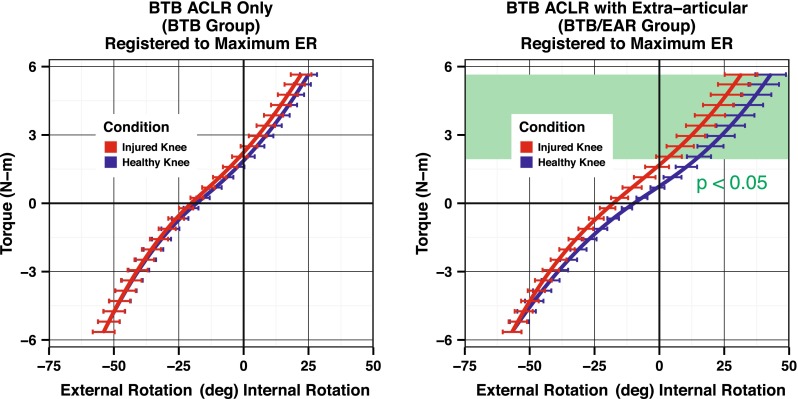
Comparison of the reconstructed and healthy knees in each group demonstrates the amount of reduction in internal rotation caused by the lateral extraarticular reconstruction

Care must be taken when interpreting these results as they represent both the extent of rotation and the initial position of the tibia with respect to the femur. The loss of external rotation demonstrated by these results is best interpreted as a repositioning of the tibia into external rotation. When this is taken into account, a better picture of the reduction in internal rotation with the lateral EAR is seen (Fig. 7). The addition of a lateral EAR limits internal rotation by 11.4° (*p* < 0.02) when compared to the healthy leg.

#### Between-group comparison

**Fig. 8:**
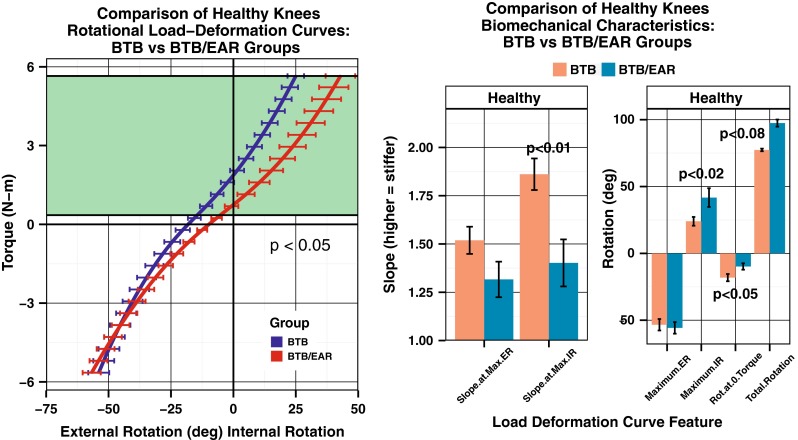
(*Left*) Average load deformation curves for the healthy knees in both groups show increased internal rotation in the healthy legs of the BTB/EAR group when compared to the BTB group. (*Right*) A comparison of features of the load deformation curves in the healthy knees of the BTB and BTB/EAR groups. Comparisons for the slope at maximum external rotation (ER) and internal rotation (IR) are shown as well as the position at maximum external rotation and internal rotation. Total rotation (ER + IR) and the rotation at zero torque are also shown

When using the rotational load deformation curves to compare the healthy legs between groups, there was a significant increase in maximum internal rotation in the BTB/EAR group (*p* < 0.05) (Fig. 8). If the injured/reconstructed legs were compared, the significant difference at maximum internal rotation disappeared (*p* < 0.10). Similarly, the healthy leg in patients in the BTB/EAR group had a significantly more compliant or softer endpoint in internal rotation, greater maximum internal rotation, and more internal rotation at torque 0 when compared to the healthy leg in patients in the BTB group (*p* < 0.05) (Fig. 8). These results show that the healthy knees in the BTB/EAR group are naturally more loose than the healthy knee in the BTB group. These differences in compliance and internal rotation between the BTB group and BTB/EAR group that were seen in the healthy knees were not evident in the reconstructed knees. The only identifiable significant difference between the reconstructed legs of the two groups was rotation at zero torque (*p* < 0.05).

## Discussion

The most important finding in this study was that the robotic testing device could identify a change in rotation of the lower leg created by the lateral extra-articular reconstruction 9 years after the reconstruction. This change was identified as a reduction in lower leg external rotation and is explained by the ‘pre-positioning’ of the tibia into 8.77° of external rotation after extra-articular reconstruction. This ‘pre-positioning’ of the tibia results in a 11.4° reduction in internal rotation seen at the leg when performing a true comparison of rotational extent between the injured leg and the healthy leg.

**Fig. 9:**
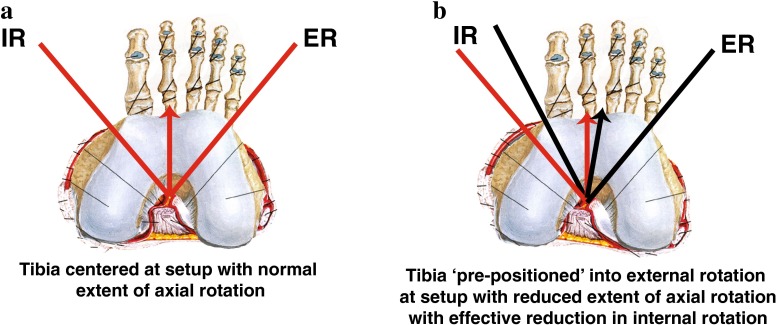
Changing the connection between the tibia and the femur by reducing internal rotation and ‘pre-positioning’ the intercalary tibia results in external rotation loss when measured at the foot. The *red arrow* represents the zero position in the healthy knee, while the *black arrow* represents the new zero position of the pre-positioned tibia after extra-articular reconstruction. The *red lines* represent the extent of internal and external tibial rotation in the healthy knee, and the *black lines* represent the new extent of tibial rotation after extra-articular reconstruction

It is important, at this point, for the reader to consider that the measurements taken represent the position of the dorsiflexed foot. The foot is rotated until it locks the tibia causing it to be rotated. The tibia then rotates until it locks with the femur. The position of the tibia in the toes-up position determines the range and position of motion seen at the foot. If the intention of the lateral EAR is to prevent internal rotation, then the tibia must be pre-positioned into external rotation relative to the foot. Thus, if the tibia is externally rotated by the EAR, then the foot will actually record a reduction in external rotation when there is actually a reduction in internal rotation between the tibia and the femur. The tibia is in effect ‘re-positioned’ into external rotation at the toes-up position creating a situation where foot moves into a reduced amount of external rotation before it reaches its normal maximum at 5.65 Nm. Taking this ‘re-positioning’ into account by registering each ‘injured’ curve to its concomitant ‘healthy’ counterpart, allows the significant reduction in internal rotation caused by the EAR to be demonstrated (Fig. 9).

The second most important finding in this study was that patients in the BTB/EAR group had significantly different rotational characteristics in their healthy, uninjured limb than those in the BTB. These findings suggest that the criteria used by the operating surgeon to select patients for an extra-articular reconstruction led him to select patients whose internal rotational endpoint had reduced stiffness (i.e. a softer endpoint), whose tibia rotated internally on average more than the group chosen for the BTB group, and whose 0 torque position was more internally rotated than the in the BTB group.

Lateral EAR in this study produced a leg with a stiffer internal rotational endpoint, in addition to a reduction in the amount of internal rotation at 5.65 Nm of torque. This is similar to a study by Monaco and colleagues, who found a significant reduction in internal rotation at 30° of knee flexion for lateral tenodesis patients compared with double-bundle reconstruction patients using intraoperative navigation [9]. While lateral extra-articular procedures have been criticized in the past for over-constraining the knee, it is difficult to perceive of an effective surgical procedure that does not have some effect on the constraint of motion in the intended direction [7]. Remember that care was taken at the time of surgery to avoid fixing the lateral tenodesis in external rotation, as described in the surgical technique. The complexity of a surgical procedure whose intent is to increase endpoint stiffness while not decreasing endpoint limitation would be difficult to predictably execute in the human body.

While there was no difference between the groups of surgically reconstructed legs based upon validated subjective outcome measures, each group may have had a different starting point. Those patients chosen by the surgeon to receive the extra-articular reconstruction appear to have naturally ‘looser’ joints. Perhaps outcomes would have been different had this group not had the additional reconstruction for their increased rotational issues.

**Fig. 10:**
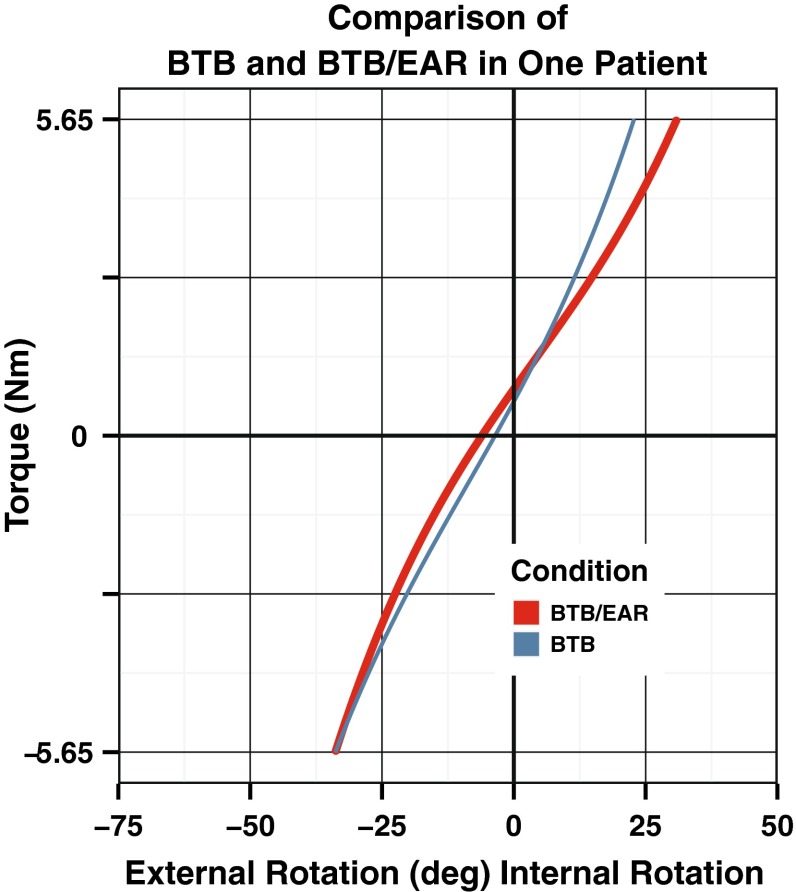
Comparison of rotational load deformation curves seen in the study patient with BTB and BTB/EAR reconstructions, identifying the impact of the extra-articular reconstruction on internal rotation characteristics

One patient had bilateral ACL reconstructions and warrants further mention. This patient’s individual rotational load deformation curves are shown in Fig. 10. Both knees are representative of a reconstructed knee. One knee had an extra-articular reconstruction (BTB/EAR) and the opposite did not (BTB). The surgeon felt that the second knee had an examination consistent with a decreased endpoint stiffness and increased internal rotation compared with the opposite knee. He chose to perform the additional extra-articular reconstruction on this patient consistent with the BTB/EAR group.

This study has a number of limitations. Firstly, the rotation at the knee was estimated using rotation of the entire lower leg. While rotation between the foot and ankle was minimized, these data are meant to compare only lower leg rotational characteristics and not knee rotational characteristics. The study examined patients who were an average of 9 years removed from surgery, so the pre-operative measurements and surgical notes were obtained retrospectively. The limitations associated with all retrospective studies apply. However, the patient questionnaires and robotic testing were applied at the time of review. In addition, the sample size of the study is small and made up of patients volunteering to undergo robotic testing. Care should be taken when applying these data to isolated and identifiable rotational characteristics at the knee. The study was able to demonstrate statistically significant results due to the precision of the robotic device. In the future, larger studies of consecutive patients are required to confirm these results.

## Conclusion

In this group of patients who were at an average of 9 years from surgery, the addition of a lateral extra-articular reconstruction to a standard bone–tendon–bone intraarticular ACL reconstruction does reduce internal rotation of the tibia with respect to the femur when compared to intraarticular reconstruction alone. It appears that the selection process for inclusion into the BTB/EAR group included an increase in total axial rotation of the healthy knee during the examination along with a decrease in endpoint stiffness at maximum internal rotation.
